# Effect of Emitted Wavelength and Light Guide Type on Irradiance Discrepancies in Hand-Held Dental Curing Radiometers

**DOI:** 10.1155/2013/647941

**Published:** 2013-10-10

**Authors:** Atsushi Kameyama, Akiko Haruyama, Masako Asami, Toshiyuki Takahashi

**Affiliations:** ^1^Division of General Dentistry, Tokyo Dental College Chiba Hospital, 1-2-2 Masago, Chiba 261-8502, Japan; ^2^Division of General Dentistry, Department of Clinical Oral Health Science, Tokyo Dental College, 2-9-18 Misaki-cho, Chiyoda-ku, Tokyo 101-0061, Japan; ^3^Division of Biomaterials Research, Oral Health Science Center, Tokyo Dental College, 2-9-18 Misaki-cho, Chiyoda-ku, Tokyo 101-0061, Japan

## Abstract

The purpose of this study was to determine any discrepancies in the outputs of five commercial dental radiometers and also to evaluate the accuracy of these devices using a laboratory-grade spectroradiometer. The power densities of 12 different curing light sources were repeatedly measured for a total of five times using each radiometer in a random order. The emission spectra of all of the curing light sources were also measured using the spectroradiometer, and the integral value of each spectrum was calculated to determine the genuine power densities, which were then compared to the displayed power densities measured by the dental radiometers. The displayed values of power density were various and were dependent on the brand of radiometer, and this may be because each radiometer has a different wavelength sensitivity. These results cast doubt upon the accuracy of commercially available dental radiometers.

## 1. Introduction

Light curing units are currently essential equipment for daily clinics in dentistry. The high reliability of direct composite restoration means that its use has expanded to be applied not only to anterior teeth but also to the posterior teeth [1]. Light curing units should also be used for the hardening of luting agents for indirect ceramic restoration [2] or for the activation of tooth whitening agents [3, 4].

Quartz-tungsten-halogen (QTH) light-curing units have been widely used in dental clinics [5]. Almost all of these units emit blue light in the 380–510 nm wavelength range to initiate camphorquinone effectively [6–8]. Since the beginning of this century, cordless light-curing units with blue light emitting diodes (LEDs) have been increasingly widely used in the dental market [9, 10] and have continued to evolve and diversify. 

It is important to emit the curing light with high power density and at a suitable wavelength. Insufficient light curing can cause degradation of the adhesive interface [11, 12], color changes in the material [13, 14], and cell cytotoxicity [15]. Sufficient light power is also required for indirect ceramic restoration, because the luting composite material must be light cured through the restored ceramic [16]. Degradation of the light source, reflector or internal filters, autoclaving of the light guide, adhesion of remnants of previously exposed restorative materials, and exposure of the light guide to disinfectant materials can significantly reduce the output intensity of the unit [17–20]. Therefore, it has been recommended that the performance of these light-curing units should be evaluated [21].

Several dental radiometers for output power density measurements are commercially available and are practical and easy to operate. However, the structures of the conversion of light into electric current are differed in each radiometer [22, 23]. The displayed power density value may therefore be different for each radiometer, even if the same light source is being measured.

The purpose of this study was to determine any discrepancies in the displayed power density of five commercial dental radiometers and also to evaluate their accuracy using a laboratory-grade spectroradiometer. The null hypothesis of this study was that there is no significant difference in the displayed output values when the same light source is emitted.

## 2. Materials and Methods

### 2.1. Light-Curing Units

As shown in Table 1, six brands of light-curing units were used in this study. Among these units, two different light guide shapes were investigated for the D-Lux 2000 (Dentrade Dental Supply & Trade, Osaka, Japan) and the Demi Plus (Kerr Hawe, Middleton, WI, USA). Three and two different types of curing modes were investigated for the G-Light Prima (GC, Tokyo, Japan) and the Bluephase G2 (Ivoclar Vivadent, Schaan, Liechtenstein), respectively.

### 2.2. Measurement of Emission Spectra

Emission spectra for each light source were measured in random order using a laboratory-grade spectroradiometer (USR-45, Ushio, Tokyo, Japan), which had recently undergone routine manufacturer maintenance and calibration. The guide tip of light-curing unit was contacted to the 6 mm-diameter aperture of light detector and fixed them by hand pressure. Subsequently, light output was detected and recorded by exclusive analytical software. The measurements were repeated five times, and three different distributions of the power densities (mW/cm^2^) were calculated from the values of the integrated value in 200–800 nm, 380–525 nm, and 430–490 nm ranges using the analytical software. 

### 2.3. Measurement of Light Intensities

The power densities of each of the light sources were also measured using five models of hand-held dental radiometers, as shown in Table 2. The measurements were randomly repeated five times and the displayed peak values of each measurement were recorded.

### 2.4. Statistical Analyses

The data obtained were statistically evaluated by analysis of variance (ANOVA) and Tukey's HSD multiple comparisons at a significance level of 0.05 in each light source. All analyses were carried out using IBM SPSS 18 (IBM Japan Inc., Tokyo, Japan).

## 3. Results

### 3.1. Emission Spectra of Light Sources

**Figure 1:**
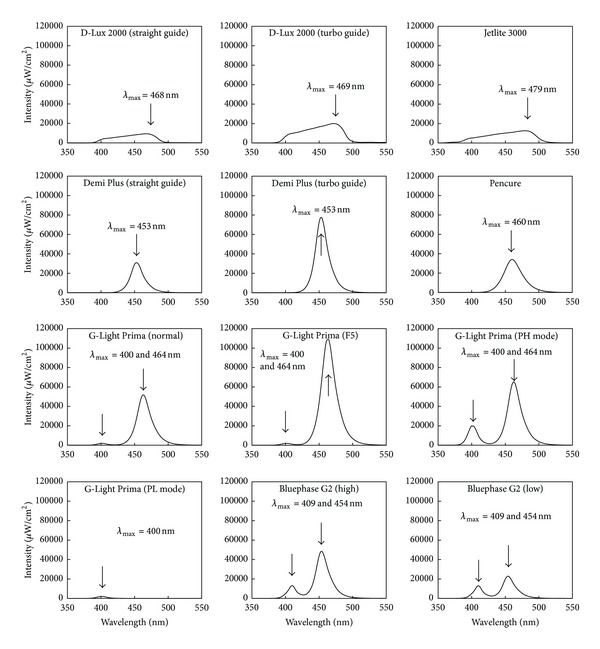
Spectral distributions of the 12 curing light sources used in this study.

The emission spectra of each of the light sources measured by the laboratory-grade spectroradiometer are shown in Figure 1, and the power densities calculated from the integrated value of each spectrum are shown in Table 3. Typical broadband spectra were shown by the two brands of QTH light source (D-Lux 2000 and Jetlite 3000, J. Morita USA, Mason Irvine, CA, USA, resp.). Both Demi Plus and the Pencure (J. Morita, Tokyo, Japan) had single-peak spectra and they had narrower bandwidths than the QTH light sources. Dual peak narrow bands in the blue and violet visible light region could be seen in both G-Light Prima (except for the PL mode) and the Bluephase G2. Almost all emissions showed a concentration in the wavelength range between 380 and 525 nm in all light sources measured in this study, except for the PL mode in the G-Light Prima.

### 3.2. Power Densities of Light Source

The power densities of each light source obtained from the laboratory-grade spectroradiometer and the five dental radiometers are shown in Table 4. The highest values were obtained in the power densities calculated from the spectroradiometer measurements rather than those of the hand-held dental radiometers for all light sources.

## 4. Discussion

Before comparison of the commercially available dental radiometers, the emissions from each light source were measured using the USR-45 laboratory-grade spectroradiometer, and the output power densities were calculated from the integrated values of each spectrum. Although the spectral distribution was different in each light source, more than 90% of the emitted light was distributed in the wavelength range between 380 and 525 nm in each case, except for the PL mode in the G-Light Prima. The calculated value from the integrated value at 380–525 nm was therefore regarded as the standard output power density to enable comparison of the displayed output power densities obtained from the five dental radiometers.

The D-Lux 2000 and Jetlite 3000 are typical QTH light-curing units (LCUs) for the polymerization of visible-light cured dental restorative materials [24]. Their emission range is from 390 to 500 nm, with a peak wavelength of 470–480 nm. Because the absorbance wavelength of camphorquinone (CQ), which is added to most light-cured restorative materials, has been reported as 430–490 nm with a peak at 468 nm, the emission ranges of these LCUs are likely to be optimal for CQ [25, 26]. D-Lux 2000 can be selected with either a turbo light guide or a conventional straight guide. Mean power densities obtained in the turbo guide were 1.83 times higher than those obtained in the straight guide. The tip diameters of the input and output sides in the turbo guide are 11 mm and 8 mm, respectively. These results were theoretically accepted because contraction of guide tip can be focused on the light power, thereby enhancing the power density by 1.89 times in theoretical that of the straight guide. 

The Demi Plus is an LED LCU that has a peak wavelength of 453 nm. This LCU has a unique technology called “periodic level shifting”, that shifts the output intensity multiple times throughout the curing cycle, and this prevents the generation of excessive heat caused by a continuous high output. This study regarded the highest displayed value as being the measured power density. As with the D-Lux 2000, we compared the output power density for straight- and turbo-light guides. The tip diameters of the input and output sides are 11 mm and 7 mm, respectively, in the turbo guide. Therefore, the theoretical increase in the power density is approximately 2.46 times that of straight guide in this case. This also agreed with the result that the mean power density when using the turbo guide was 2.40 times that when using the straight guide, when measured by the USR-45.

The Pencure is a single-peak LED LCU, similar to the Demi Plus. In contrast, the G-Light Prima and the Bluephase G2 are “dual-wave” LCUs that contain not only blue LEDs but also supplementary violet LED(s) to polymerize 1-phenyl-1,2-propanedione (PPD) and 2,4,6-trimethylbenzoyl diphenyl-phosphine oxide (Lucirin TPO) effectively [25, 26]. For the Pencure and the G-Light Prima, the displayed power densities in the Bluephase Meter and the Cure Rite were approximately 30% and 25% lower, respectively, than those of the USR-45 spectroradiometer. When the Demi Plus and the Bluephase G2 were measured, on the other hand, the displayed values of the Cure Rite were rather lower than those of the Bluephase Meter. The spectral results showed that the peak wavelengths of the Pencure and the G-light Prima were shifted 7–10 nm to the right of those of the Demi Plus and the Bluephase. Therefore, we deduced that the Cure Rite might be more sensitive to light emissions at 460–465 nm and less sensitive to those at about 450 nm. Conversely, the Bluephase Meter might be more sensitive at about 450 nm and less sensitive in the 460–465 nm range. 

For the G-Light Prima, the F5 operating mode enhances the emission of blue LEDs in comparison with the normal mode. The power density measured by the USR-45 was found to be 1.56 times higher in the F5 mode than in the normal mode, and the increase was almost the same as that measured by the Bluephase meter (1.57 times). However, the increase measured by the Curing Light Meter 105 was different to that of the USR-45 spectroradiometer. Because this dental radiometer displays the measured values on an analog scale with irregular scale intervals and a 3D-curved scale bar, it was difficult to determine the light output precisely. 

The PH mode of the G-Light Prima enhances the output intensities of the violet LED in comparison with the normal mode, although the blue LED output is almost the same as in the normal mode. The output of the PH mode measured using the USR-45 indicated that it was about 50 mW/cm^2^ higher than that of the normal mode, and this may be caused by the enhancement of the violet LED. However, the displayed output values in the Bluephase Meter, the Curing Light Meter 105, and the Model L.E.D. radiometer were rather lower in the PH mode than in the normal mode. This therefore suggested that these radiometers could not detect the subtle violet light irradiated in the PL mode, although 50 mW/cm^2^ violet light was emitted. This therefore showed that the commercial dental radiometers investigated in this study could not be used to compare the precise output power density for different emission modes [22, 23].

The Model 100 Optilux Radiometer and the Model L.E.D. Radiometer are produced by the same manufacturer, and their mechanisms are similar apart from their measurement output limits [23]. The radiometer units used in this study were ordered after the initial planning of this research and were used for the first time during this research. Nevertheless, we found that the displayed output values of the Model 100 Radiometer tended to be about 20% higher than those measured by the Model L.E.D. radiometer. It is not possible to calibrate the displayed power density in almost all of these commercially available dental radiometers. Therefore, the results suggest that the reliability of a commercial dental radiometer might depend on its calibration condition before shipping.

Insufficient output intensities and incompatibility between the curing light wavelengths and the absorption of the photo initiators might cause degradation of the cured materials themselves [27–29], thereby leading to a reduction in clinical reliability. Therefore, frequent confirmation of the LCU light output before use on the patient is recommended. However, the results of this study cast doubt upon the accuracy of many commercially available dental radiometers. It is will thus be necessary to develop a new dental radiometer which is able to measure any type of LCU, to be calibrated precisely, and to be manipulated easily beside the patient.

## 5. Conclusion

This study investigated the discrepancies in the outputs of five commercially available dental radiometers and compared their results to those measured using a laboratory-grade spectroradiometer. The displayed output values were various and depended on the brand of the radiometer. These results might be caused by each of the radiometers having different wavelength sensitivities. 

## Figures and Tables

**Table 1:** Curing light sources used in this study.

Light-curing unit (Serial no.)	Manufacturer	Type	Light guide	Curing mode
D-Lux 2000 (JB1646)	Dentrade Dental Supply & Trade, Osaka, Japan	QTH	Straight Turbo (11/8 mm)	High
Jetlite 3000 (9040763)	J. Morita USA, Mason Irvine, CA, USA	QTH	Straight	High
Demi Plus (760012733)	Kerr Hawe, Middleton, WI, USA	Single-peak LED	Straight Turbo (11/7 mm)	— —
Pencure (SN13444)	J. Morita, Tokyo, Japan	Single-peak LED	Turbo (11/8 mm)	—
G-Light Prima (00156)	GC, Tokyo, Japan	Dual-wave LED		Normal F5 PH PL
Bluephase G2 (209409)	Ivoclar Vivadent, Schaan, Liechtenstein	Dual-wave LED	Straight	High Low

**Table 2:** Radiometers investigated in this study.

Radiometer (Serial no.)	Manufacturer	Type	Measurable wavelength	Measurable output intensity
USR-45 (—)	Ushio, Tokyo, Japan	Labo-grade	200–800 nm	—
Bluephase Meter (005386)	Ivoclar Vivadent, Schaan, Liechtenstein	Digital	380–515 nm	300–2500 mW/cm^2^
Cure Rite (7781)	Dentsply Caulk, Milford, DE, USA	Digital	400–525 nm	<2000 mW/cm^2^
Curing Light Meter 105 (4237/2009)	Rolence Enterprise, Chingli, Taiwan	Analogue	400–500 nm	<2500 mW/cm^2^
Model 100 Optilux Radiometer (145975)	SDS Kerr, Middleton, WI, USA	Analogue	400–500 nm	<1000 mW/cm^2^
Model L.E.D. Radiometer (79310406)	SDS Kerr, Middleton, WI, USA	Analogue	400–500 nm	<2000 mW/cm^2^

**Table 3:** Power density calculated from integral value of spectra at 200–800 nm, 380–525 nm, and 430–490 nm (*n* = 5, mW/cm^2^).

Light-curing unit	Light guide/mode	200–800 nm	380–525 nm	430–490 nm
D-Lux 2000	Straight guide	790 ± 54	728 ± 41	509 ± 28
		(100%)	(92.2%)	(64.4%)
	Turbo guide	1450 ± 56	1336 ± 51	940 ± 37
		(100%)	(92.1%)	(64.8%)

Jetlite 3000	—	1016 ± 57	966 ± 56	613 ± 32
		(100%)	(95.0%)	(60.3%)

Demi Plus	Straight guide	837 ± 6	811 ± 5	781 ± 5
		(100%)	(96.9%)	(93.3%)
	Turbo guide	1997 ± 58	1947 ± 56	1881 ± 55
		(100%)	(97.5%)	(94.1%)

Pencure	—	1275 ± 18	1236 ± 17	1139 ± 16
		(100%)	(96.9%)	(89.3%)

G-Light Prima	Normal	2121 ± 68	2068 ± 66	1922 ± 58
		(100%)	(97.5%)	(90.6%)
	F5	3334 ± 85	3238 ± 82	2991 ± 75
		(100%)	(97.1%)	(89.7%)
	PH	2169 ± 100	2112 ± 100	1615 ± 101
		(100%)	(97.3%)	(74.4%)
	PL	51 ± 3	42 ± 3	0.9 ± 0
		(100%)	(82.3%)	(1.8%)

Bluephase G2	High	1528 ± 27	1489 ± 25	1215 ± 29
		(100%)	(97.4%)	(79.5%)
	Low	839 ± 5	815 ± 7	563 ± 18
		(100%)	(97.1%)	(67.1%)

**Table 4:** Displayed light output (Mean ± S.D.; mW/cm^2^) and percentage of the reduction from USR-45 (%) (*n* = 5).

	USR-45 (380–525 nm)	Bluephase Meter	Cure Rite	Curing Light Meter 105	Model 100 Optilux	Model L.E.D. Radiometer
D-Lux 2000	728 ± 41^a^	618 ± 31^b^	426 ± 27^c^	306 ± 26^d^	616 ± 32^b^	442 ± 35^c^
(Straight)	(100%)	(84.9%)	(58.5%)	(42.0%)	(84.6%)	(60.7%)
D-Lux 2000	1336 ± 51^a^	1316 ± 94^b^	682 ± 64^d^	690 ± 42^d^	980 ± 27^c^	710 ± 42^d^
(Turbo)	(100%)	(98.5%)	(51.0%)	(51.6%)	(73.4%)	(53.1%)
Jetlite 3000	966 ± 56^a^	730 ± 25^b^	543 ± 42^c^	456 ± 13^d^	688 ± 48^b^	526 ± 37^cd^
	(100%)	(75.6%)	(56.2%)	(47.2%)	(71.2%)	(54.5%)
Demi Plus	811 ± 5^a^	684 ± 25^b^	538 ± 8^d^	492 ± 11^e^	790 ± 11^a^	600 ± 0^c^
(Straignt)	(100%)	(84.3%)	(66.3%)	(60.7%)	(97.4%)	(74.0%)
Demi Plus	1947 ± 56^a^	1756 ± 83^b^	1395 ± 21^c^	1680 ± 45^b^	1000 ± 0^∗d^	1410 ± 22^c^
(Turbo)	(100%)	(90.2%)	(71.6%)	(86.3%)	(51.4%)	(72.4%)
Pencure	1236 ± 17^a^	868 ± 26^d^	917 ± 17^c^	830 ± 35^d^	1000 ± 0^∗b^	850 ± 35^d^
	(100%)	(70.2%)	(74.2%)	(67.1%)	(80.9%)	(68.8%)
G-Light Prima	2068 ± 66^a^	1702 ± 72^b^	2000 ± 0^∗a^	880 ± 27^e^	1000 ± 0^∗d^	1130 ± 27^c^
(Normal)	(100%)	(82.3%)	(96.7%)	(42.6%)	(48.4%)	(54.6%)
G-Light Prima	3238 ± 82^a^	2672 ± 143^b^	2000 ± 0^∗d^	2500 ± 0^∗c^	1000 ± 0^∗e^	1950 ± 50^d^
(F5 mode)	(100%)	(82.5%)	(61.8%)	(77.2%)	(30.9%)	(60.2%)
G-Light Prima	2112 ± 100^a^	1496 ± 23	2000 ± 0^∗b^	800 ± 0^e^	1000 ± 0^∗d^	1010 ± 22^d^
(PH mode)	(100%)	(70.8%)	(94.7%)	(37.9%)	(47.3%)	(47.8%)
G-Light Prima	42 ± 3^a^	0 ± 0^b^	0 ± 0^b^	0 ± 0^b^	0 ± 0^b^	0 ± 0^b^
(PL mode)	(100%)	(0%)	(0%)	(0%)	(0%)	(0%)
Bluephase G2	1489 ± 25^a^	1312 ± 71^b^	870 ± 3^e^	970 ± 27^d^	1000 ± 0^∗cd^	1038 ± 16^c^
(High)	(100%)	(88.1%)	(58.4%)	(65.1%)	(67.2%)	(69.7%)
Bluephase G2	815 ± 7^a^	668 ± 33^b^	406 ± 3^d^	400 ± 0^d^	700 ± 0^b^	550 ± 0^b^
(Low)	(100%)	(82.0%)	(49.8%)	(49.1%)	(85.9%)	(67.5%)

Within the same row, different letters indicate groups that are statistically different (*P* < 0.05).

*Achieved to the highest limit of radiometer concerned.
